# Acute ischemic stroke and measurement of apixaban and rivaroxaban: an observational cohort implementation study

**DOI:** 10.1016/j.rpth.2023.102307

**Published:** 2024-01-02

**Authors:** Erik Koldberg Amundsen, Hege Ihle-Hansen, Kristian Lundsgaard Kraglund, Guri Hagberg

**Affiliations:** 1 Department of Medical Biochemistry, Oslo University Hospital, Oslo, Norway; 2 Oslo Stroke Unit, Department of Neurology, Oslo University Hospital, Oslo, Norway

**Keywords:** apixaban, factor Xa inhibitors, rivaroxaban, stroke, thrombolytic therapy

## Abstract

**Background:**

Treatment with intravenous thrombolysis for acute ischemic stroke is contraindicated with intake of apixaban/rivaroxaban in the last 48 hours. Recent European Stroke Organization guidelines suggest that thrombolysis can be considered if anti-factor Xa activity (AFXa) is <0.5 × 10^3^ IU/L with low-molecular-weight (LMWH) or unfractionated heparin (UFH) calibrated assays. Some centers also use apixaban/rivaroxaban-calibrated AFXa assays to identify patients with low drug concentrations.

**Objectives:**

To prospectively evaluate the first year of implementation of drug-calibrated AFXa assays at our center with 2500 yearly admittances with suspected stroke.

**Methods:**

Samples were analyzed on Sysmex CS-5100 instruments with Innovance anti-Xa reagents. Thrombolysis could be considered with drug concentrations <25 μg/L. Patients were registered in an institutionally approved quality register. Outcomes included (1) the number of patients receiving thrombolysis after drug measurement, (2) turn-around time for drug concentration measurements, and (3) sensitivity of LMWH/UFH AFXa to apixaban and rivaroxaban.

**Results:**

Apixaban or rivaroxaban was measured in 148 samples, and 4 patients who previously would have been ineligible for thrombolysis were treated with thrombolysis. In total, thrombolysis was administered in 123 patient episodes in the study period. The median turn-around time for the drug measurements was 38 minutes. Apixaban concentrations of 25 μg/L and 50 μg/L corresponded to LMWH/UFH AFXa of 0.13 and 0.27 × 10^3^ IU/L, respectively. There were too few rivaroxaban results for regression analysis.

**Conclusion:**

Implementation of apixaban and rivaroxaban measurements led to a small increase in the number of patients receiving thrombolysis. Excluding significant concentrations of apixaban or rivaroxaban using LMWH/UFH AFXa may be feasible.

## Essentials

• Thrombolysis is contraindicated in anticoagulated patients with acute stroke due to bleeding risk.
• We introduced apixaban and rivaroxaban testing to identify patients who could get thrombolysis.
• During 1 year, 123 patients received thrombolysis and 4 extra due to low drug concentration.
• Providing rapid apixaban and rivaroxaban testing may result in a small but important benefit.

## Graphical abstract

**Figure:**
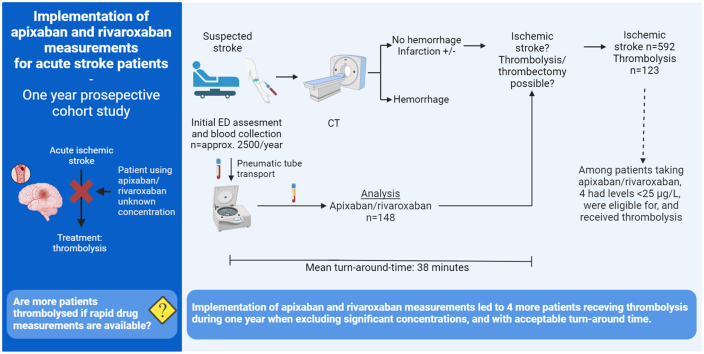

## 1 Introduction

Acute ischemic stroke (AIS) is a leading cause of death and disability worldwide, and due to an aging population, the absolute number of incident strokes is increasing [1]. Early reperfusion therapy with intravenous thrombolysis with alteplase can dramatically improve patient outcomes [2]. However, as many as 20% of patients with AIS use direct oral anticoagulants (DOACs) before the stroke event, and the use of DOACs is increasing [[3], [4], [5]]. Thrombolysis is contraindicated with recent ingestion (within 48 hours previously) due to the presumed increased risk of symptomatic intracranial hemorrhage [4,6]. Studies show that the compliance or dosage of DOACs may be insufficient [7], and the time of the last dose is often unknown or uncertain at admittance to the emergency department (ED). Thus, patients with low drug concentrations or no prior intake of DOACs can still be considered ineligible for thrombolysis if drug concentration measurements are unavailable.

The 2021 edition of the European Stroke Organization (ESO) guideline for acute treatment recommends considering thrombolysis for patients on factor Xa inhibitors such as apixaban and rivaroxaban when anti-FXa activity (AFXa) is <0.5 × 10^3^ IU/L, presumably representing an activity corresponding to low drug concentrations [6]. However, anti-FXa assays calibrated for heparin (low-molecular-weight [LMWH]/unfractionated heparin [UFH] AFXa) have variable sensitivity for apixaban and rivaroxaban. In a comparison of 3 different assays, Mithoowani et al. [8] found that 50 μg/L corresponded to LMWH/UFH AFXa of 0.28 to 0.88 × 10^3^ IU/L and 0.41 to 1.45 × 10^3^ IU/L for apixaban and rivaroxaban, respectively. Thus, it seems necessary to have a specific definition of LMWH/UFH AFXa cutoffs for each combination of drug and measurement procedure [9].

Some centers use apixaban- or rivaroxaban-calibrated AFXa assays to measure drug concentrations more directly. A recently published international, multicenter, retrospective cohort study showed standard operating procedures (SOP) for thrombolysis in patients with recent ingestion of DOACs at 49 stroke centers worldwide, with remarkably different cutoffs and selection strategies [10]. A French expert group has previously suggested that thrombolysis can be administered when apixaban or rivaroxaban concentration is <50 μg/L [11]; others have suggested no contraindication for thrombolysis if rivaroxaban concentrations are <100 μg/L [12], and apixaban <10 μg/L [13]. However, these studies lack clinical validation [8].

LMWH/UFH AFXa or apixaban- and rivaroxaban-calibrated AFXa are not routinely available at many centers. Costs, or perceptions about costs, turn-around time, clinical utility, and regulatory approval of measurement procedures, are possible barriers to implementation [14].

To “get with the guidelines,” an SOP to guide decisions for the use of thrombolysis incorporating both LMWH/UFH and apixaban- and rivaroxaban-calibrated AFXa assays were established at the Oslo stroke center, with a cutoff for thrombolysis <25 μg/L for both drugs. We prospectively evaluated the first year of implementation of the SOP using data from a local quality register. Several aims were investigated: (1) if the implementation led to more patients receiving thrombolysis, (2) the turn-around time for the measurements, and (3) the cutoff limits for LMWH/UFH AFXa compared with drug-calibrated AFXa assays.

## 2 Methods

### 2.1 Patients

Treatment of patients with suspected stroke in Oslo is centralized at Oslo University Hospital, Ullevaal. If indicated, the center gives reperfusion therapy with thrombolysis and/or thrombectomy. According to data from recent years, we assess around 2500 patients for suspected stroke each year, with around 800 diagnosed with AIS, including 300 admitted within 4 hours of symptom onset, of whom 23% receive thrombolysis with an average door-to-needle time of 28 minutes [5]. The assessment of patients in the ED with the measurement of apixaban or rivaroxaban is shown in the graphical abstract.

Apixaban and rivaroxaban are the most frequently used DOACs in Norway, accounting for 67% and 22% of patients on DOACs, respectively [3]. Based on the proportion of patients with atrial fibrillation in the stroke population, we estimated before the implementation that 20% of patients presenting with AIS would use a DOAC.

### 2.2 Recruitment

Measurement methods for apixaban and rivaroxaban were introduced on September 15, 2021, and for the first year after the implementation, all patients admitted with suspicion of stroke on apixaban or rivaroxaban were consecutively registered in a quality register. There were no exclusion criteria. We assumed that the ethnicity of the patients was similar to the population of Oslo, but this was not registered. The biomedical laboratory scientist is part of the stroke call in the ED, facilitating rapid blood collection. All stroke physicians in the ED were instructed to order apixaban/rivaroxaban and LMWH/UFH AFXa for patients using either drug. Due to the uncertainty of the cutoffs, a conservative approach for excluding clinically relevant drug concentrations was chosen with cutoffs at 25 μg/L for both drugs with the drug-calibrated assays. Patients also had to satisfy ESO clinical criteria to be eligible for thrombolysis [6]. Apixaban and rivaroxaban could also be ordered for up to 48 hours after blood collection from stored samples in patients with stroke without indication for thrombolysis and no need for immediate analysis.

### 2.3 Variables

Relevant demographic variables, stroke etiologies, use of antithrombotics, drug concentrations, complications, and stroke severity assessed by the National Institutes of Health Stroke Scale (NIHSS) [15] were collected from the electronic medical record and registered in a quality register by a dedicated study nurse and stroke physician. To ensure the inclusion of all relevant patients, the electronic medical record was checked for all patients with apixaban or rivaroxaban measurements ordered from samples in the ED during the study period, and concentrations were cross-checked. The total number of patients admitted with AIS and those treated with thrombolysis was retrieved from local quality registers.

### 2.4 Laboratory methods

Venous or arterial blood samples were collected into 3.2% citrated tubes, which were rapidly transported by a pneumatic tube system to the central laboratory and centrifuged at 2800 × g for 5 minutes before analysis in primary tubes. We have previously verified that these centrifugation conditions lead to less than 10 × 10^9^/L residual platelets in plasma. Additionally, we compared the centrifugation at 2800 × g for 5 minutes with 2500 × g for 15 minutes for 2 blood samples spiked with apixaban or rivaroxaban with acceptable results (less than 10% difference). The samples were stored in primary tubes at room temperature for up to 48 hours; previous studies have indicated that apixaban and rivaroxaban measurements (AFXa) are stable for several days [16].

Apixaban, rivaroxaban, and LMWH/UFH AFXa analyses were performed on Sysmex CS-5100 instruments. For the apixaban and rivaroxaban assays, AFXa was converted to drug concentration using a calibration curve derived from apixaban and rivaroxaban calibrators, respectively. We used the Innovance anti-Xa reagents (Siemens Healthineers) for all 3 methods. Until June 13, 2022, we used Biophen Apixaban/Rivaroxaban Calibrators (Hyphen Biomed) and an instrument application made for the Biophen assay. Since June 14, 2022, we have used the new Siemens *in vitro* diagnostic regulation approved apixaban/rivaroxaban calibrators and applications. Further details about the laboratory methods are described in Supplementary Tables S1 and S2. When comparing the methods used before and after June 14, 2022, the differences were found to be acceptable (Supplementary Figures S1 and S2). Daily internal quality control was performed, and performance in external quality assessment was acceptable during the study period. The turn-around time was calculated from the time of blood collection to the time of the electronic report of the results from the laboratory information system. All orders with a turn-around time of more than 1 hour were manually reviewed, and orders where apixaban/rivaroxaban was requested more than 30 minutes after blood collection were not included in the calculation of turn-around time. In this case, we considered that apixaban/rivaroxaban was ordered not to consider eligibility for thrombolysis but to compare to LMWH/UFH AFXa and management of the patient in the diagnostic follow-up.

### 2.5 Total yearly cost of drug measurements

The cost for reagents, calibrators, and controls was obtained from Siemens Healthineers. The company does not report list prices, but the stated prices are representative of the Nordic market. We assumed that calibration is performed once a year and that 2 apixaban controls and 2 rivaroxaban controls are run every day. The costs of calibration and controls will be the same regardless of the number of ordered analyses per year. Additionally, we estimated a yearly cost of 20 working hours for a biomedical laboratory scientist to run internal and external quality controls and various tasks necessary to be able to report apixaban and rivaroxaban. The marginal cost for working hours when running an apixaban or rivaroxaban analysis in addition to International Normalized Ratio and activated partial thromboplastin time, which would still be analyzed, was considered negligible. For the currency conversions, we used the average exchange rate for 2021 (1 Norwegian krone = €0.0984, 1 USD = €0.846).

### 2.6 Statistical analysis

Continuous variables are presented as mean ± SD if they appeared to be normally distributed by visual inspection or otherwise as median with IQRs. Categorical variables are presented as numbers and percentages (%). Simple linear regression was performed in GraphPad Prism 9.4.1 (GraphPad Software). Missing data for LMWH/UFH AFXa were assumed to be missing at random and not imputed. Figures were created with GraphPad Prism 9.4.1 or BioRender.com.

### 2.7 Ethical considerations

The decision to implement the apixaban and rivaroxaban measurements was based on a clinical need to meet the new acute reperfusion treatment options recommended by the ESO guidelines [6]. However, this depended upon reliable concentration measurements and monitoring of the implementation to ensure its safety and effectiveness. Thus, patients were registered in a quality register approved by the head of the Stroke Department. By Norwegian law, patient consent is not necessary or mandatory for quality improvement projects. The register was approved by the hospital’s Data Protection Officer (reference number 21/11742).

## 3 Results

### 3.1 Patient characteristics

During the 1-year follow-up period, we registered 148 episodes from 139 patients admitted with suspected stroke with known use of apixaban (124 episodes) or rivaroxaban (24 episodes). Of these, 72 episodes (49%) were diagnosed as an AIS. The most frequent indication for anticoagulation was atrial fibrillation. Patient characteristics are shown in Table 1. During the study period, there were 592 episodes with AIS; in 123 (20.8%) episodes, patients were treated with thrombolysis.

**Table 1:** Patient characteristics in patients admitted with suspected stroke.

Characteristic	*n* (%)^a^
Age, years (SD)	80.8 (10.1)
No. of episodes	148
No. of patients	139
Women	59 (40)
**Indication DOAC (% per episode)**	
Venous thrombosis	11 (7.4)
Atrial fibrillation	132 (89)
Other/unknown	5 (3.3)
**Diagnosis (% per episode)**	
Ischemic stroke	72 (49)
Brain hemorrhage	18 (12)
Other	58 (39)
**Thrombolysis**	4 (2.7)
**Drug concentration (mean, SD)**	
Apixaban μg/L (*n* = 124, 2 had missing results)	133 (73)
Rivaroxaban μg/L (*n* = 24, 1 had missing results)	181 (139)
**Hours since last apixaban/rivaroxaban dose**	
Dose 1 time a day (median, IQR)	8.0 (4.3-16.2)
Dose 2 times a day (median, IQR)	7.7 (5.0-12.0)
Unknown time of last dose	7 (4.8)

DOAC, direct oral anticoagulant.

a Unless otherwise specified.

### 3.2 Apixaban and rivaroxaban results, treatment with thrombolysis, and turn-around time

**Figure 1:**
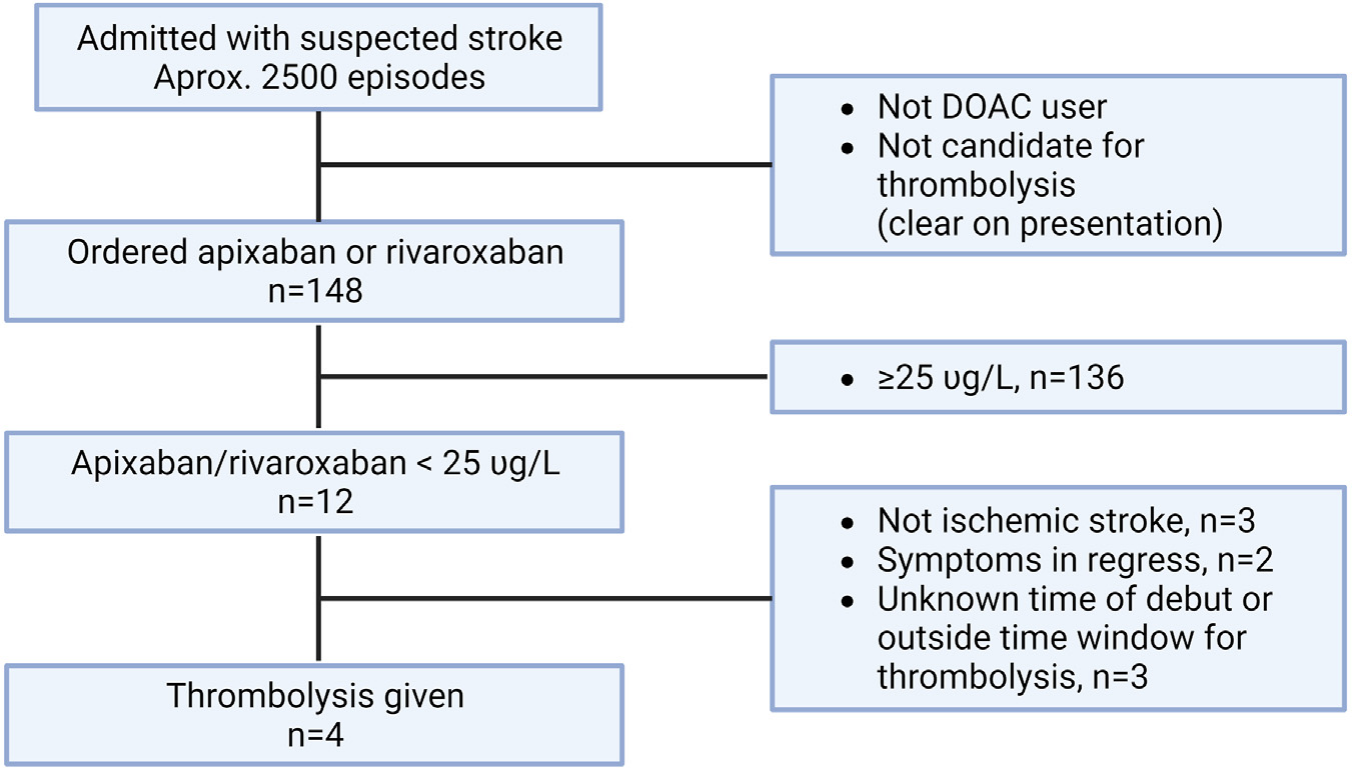
Overview, inclusion, assessment, and treatment of patients. Created with BioRender.com. DOAC, direct oral anticoagulant.

**Figure 2:**
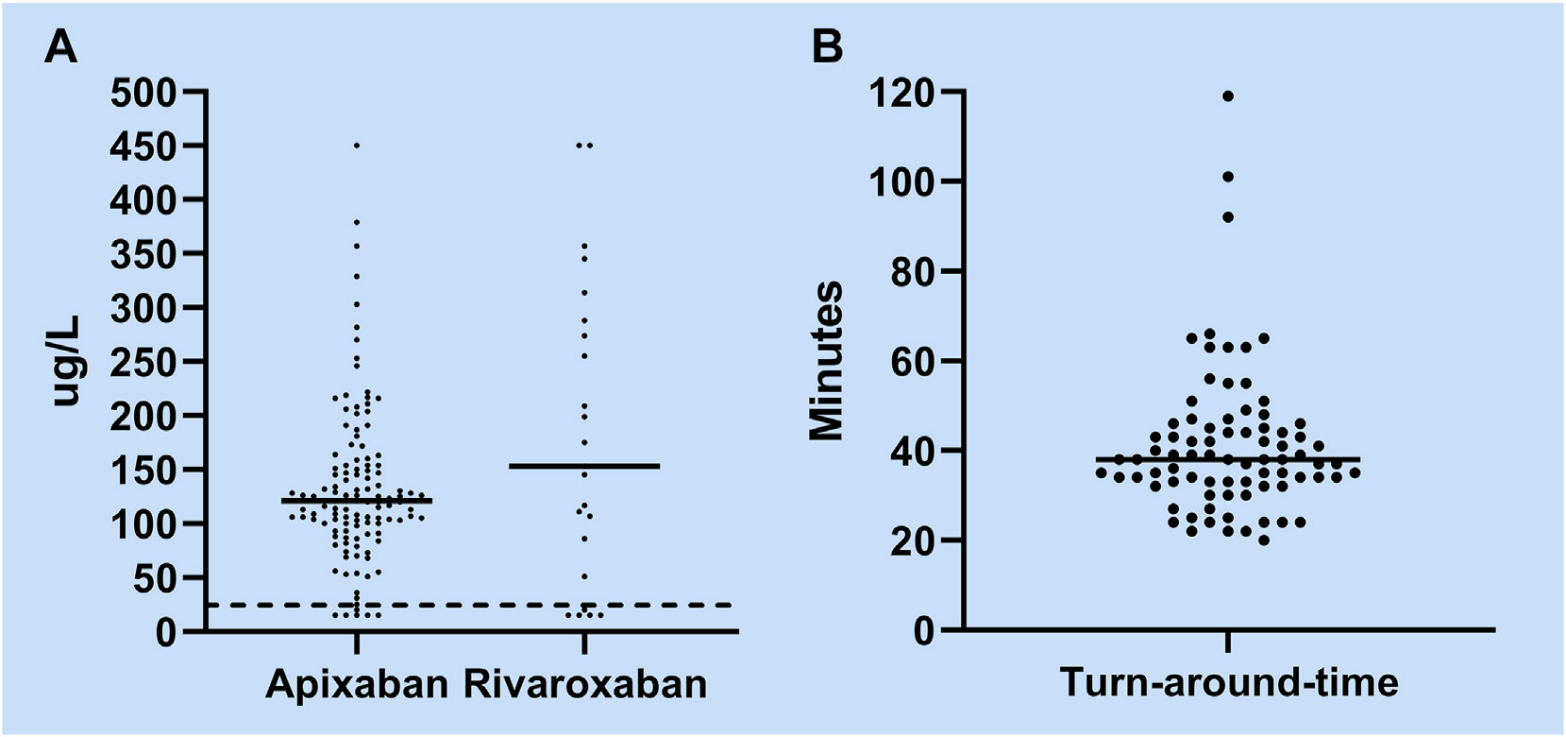
(A) Apixaban and rivaroxaban concentrations. The dashed line at the cutoff is 25 μg/L, and the full lines represent the medians. (B) Turn-around time for apixaban and rivaroxaban measurements ordered from the Emergency Department; the full line represents the median.

The inclusion and treatment of patients is shown in Figure 1. Drug concentrations were missing for 2 patients using apixaban and 1 patient using rivaroxaban. The majority of patients had drug concentrations in the range of 100 to 200 μg/L (Figure 2A). Seven and 5 patients had apixaban or rivaroxaban below the cutoff of 25 μg/L, respectively. Of these, 1 patient on apixaban and 3 on rivaroxaban were otherwise eligible and were treated with thrombolysis (Table 2). The other 8 patients had an unknown time for the stroke onset, were admitted too late, or had symptoms in regression. None of the 8 patients with a known time of last drug intake and drug concentration below 25 μg/L had taken the last dose more than 48 hours ago (range, 7 to 32 hours). Of the 4 patients with apixaban/rivaroxaban concentration below the cutoff of 25 μg/L treated with thrombolysis, 2 had a marked clinical improvement according to the NIHSS (1 also had a thrombectomy); 1 patient with severe symptoms had no improvement, and 1 had a severe intracerebral hemorrhage (ICH) complication and died. Retrospectively, the medical history of this last patient changed from an unknown intake of DOAC to discontinuation of apixaban several weeks before the stroke.

**Table 2:** Characteristics of patients treated with intravenous thrombolysis.

Patient no. (drug)	Time, symptoms debut to admittance (h:min)	Time since the last apixaban or rivaroxaban dose (h:min)	TAT (min)	DTNT (min)	NIHSS admittance	NIHSS 24 hours	Complications
1 (apixaban)	0:40	Unknown	33	51	23	Dead	Cerebral bleeding
2 (rivaroxaban)	2:22	32:00	43	46	24	24	None
3 (rivaroxaban)	1:02	08:45	28	54	7	1	None
4 (rivaroxaban)^a^	1:00	Unknown	79	125	8	0	None

DTNT, door-to-needle time; NIHSS, National Institute of Health Stroke Scale; TAT, turn-around time.

a Treated with thrombectomy following intravenous thrombolysis.

The number of patients eligible at alternative cutoffs at 50 μg/L and 100 μg/L are shown in Table 3.

**Table 3:** Number of patient episodes eligible for thrombolysis in different apixaban or rivaroxaban concentration intervals (*N* = 148 patient episodes).

Episodes	Drug concentration, *n* (%)		
	<25 μg/L	≥25 and <50 μg/L	≥50 and <100 μg/L
Episodes	12 (8)	3 (2)	25 (17)
Episodes, otherwise eligible for thrombolysis	4 (3)	0 (0)	12 (8)

The median turn-around time was 38 minutes (IQR, 33-46) (Figure 2B). The turn-around time for the 4 patients who were treated with thrombolysis is shown in Table 2. No patients were denied thrombolysis due to delayed apixaban or rivaroxaban results.

### 3.3 Comparison apixaban/rivaroxaban and low-molecular-weight/unfractionated heparin anti-factor Xa

**Figure 3:**
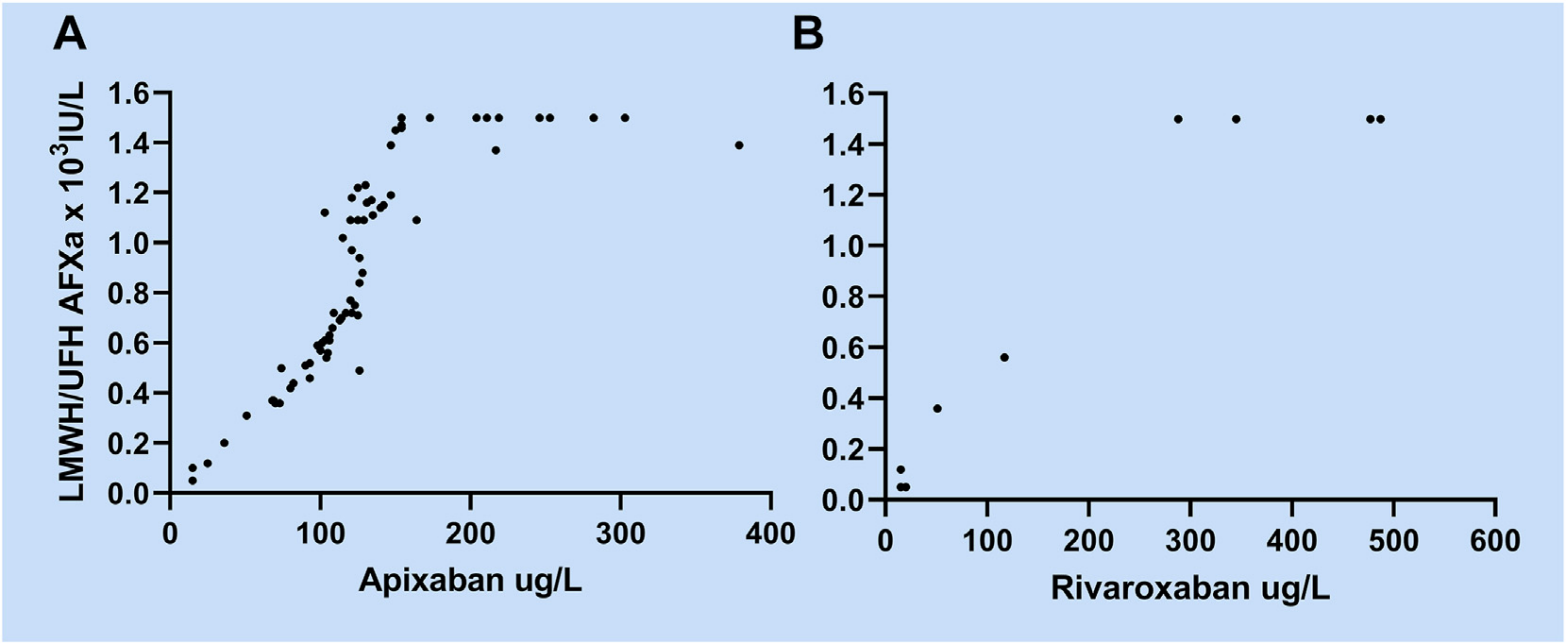
Comparison of apixaban (A, *n* = 71) and rivaroxaban (B, *n* = 11) results with low-molecular-weight or unfractionated heparin anti-factor Xa activity (LMWH/UFH AFXa).

LMWH/UFH AFXa results were available for 84 patients; 72 were on apixaban, and 12 were on rivaroxaban. Of these, 14 (19%) and 6 (50%) were below 0.5 × 10^3^ IU/L. Two patients with LMWH/UFH AFXa were missing results for apixaban or rivaroxaban. The comparisons of apixaban and rivaroxaban concentrations with LMWH/UFH AFXa are shown in Figure 3.

Using linear regression analysis of the samples with apixaban concentration ≤100 μg/L, the cutoff for LMWH/UFH AFXa corresponding to 25 μg/L and 50 μg/L were found to be 0.13 and 0.27 × 10^3^ IU/L, respectively. The ESO recommended cutoff at 0.5 × 10^3^ IU/L corresponded to 90 μg/L for apixaban. For rivaroxaban, the number of results was not sufficient to perform regression analysis.

### 3.4 Total yearly cost of drug measurements

The total yearly costs of apixaban and rivaroxaban measurements were €8124 and €7792, respectively (Supplementary Table S3). Approximately 75% of the costs were for controls, calibration, and other work necessary to be able to report apixaban and rivaroxaban results.

## 4 Discussion

We introduced a local SOP for patients admitted with suspected stroke, incorporating apixaban- and rivaroxaban-calibrated AFXa assays in clinical decision-making. In the first year after implementation, this led to thrombolysis of 4 patients with AIS who previously would have been ineligible out of 123 episodes of patients treated with thrombolysis. The median turn-around time was 38 minutes, showing that measurements are feasible in a time-dependent acute setting. The sensitivity of the LMWH/UFH AFXa assay to apixaban was sufficient to use the method for the exclusion of clinically relevant apixaban concentrations. We could demonstrate a linear relationship between apixaban and LMWH/UFH AFXa results below 100 μg/L.

The percentage of patients with suspected stroke using apixaban or rivaroxaban was lower than previously reported in a Swiss register study [4]. The cutoff for the safe administration of thrombolysis is not well established [6,7,[11], [12], [13]], and we thus chose a low cutoff (25 μg/L). With a higher cutoff, more patients would have been eligible for thrombolysis. The ESO suggests that thrombolysis can be given when AFXa is below 0.5 × 10^3^ IU/L, which corresponds to a drug concentration of 90 μg/L for apixaban. A recent large-scale register study showed that some centers use a cutoff at 100 μg/L [10]. Using this cutoff, 12 more patients would have been eligible for thrombolysis compared to the 25 μg/L cutoff. Interestingly, this study also indicates that patients with AIS on DOACs have a lower risk of procedure-related ICH than patients who do not use DOACs, irrespective of the drug concentration [10]. However, selection bias could explain this finding, as patients on DOACs with high expected bleeding risk did not receive thrombolysis. Patients with atrial fibrillation on DOACs are generally older and with more comorbidities, with an increased risk of bleeding complications after thrombolysis compared with the general stroke population. Further, the mean NIHSS in our study population and the Norwegian Stroke Registry is lower than in the referred study, indicating milder strokes [5,10], where potential treatment effects must be balanced with the increased bleeding risk. The safety of thrombolysis for patients on DOACs should be validated in prospective studies. It is also important to note that similar concentrations of the different DOACs may not result in similar anticoagulant effects [17].

Interestingly, the patients with apixaban or rivaroxaban below the cutoff had relatively short time intervals since the last dose, much shorter than the 48 hours indicated as the time limit for safe thrombolysis treatment according to current guidelines. This indicates that drug concentration measurements are also helpful when the time of the last dose is known to be within the last 48 hours.

A sufficiently short turn-around time is necessary to ensure that the results will be useful in clinical decision-making, as thrombolysis efficiency is highly time-dependent. At our institution, the biomedical laboratory scientist assesses the patient in the ED as part of the stroke call, and we already had a rapid transportation system with high priority for samples from this patient group. It is important to note that each laboratory must verify that the centrifugation conditions are adequate. The door-to-needle time for the 4 patients who were treated with thrombolysis was longer than desirable and longer than the average at our institution (28 minutes). The risk of bleeding complications must outweigh the risk of poor outcomes due to treatment delay. This could be improved if point-of-care tests become available.

Using LMWH/UFH AFXa instead of the apixaban- or rivaroxaban-calibrated assays is an attractive option from the laboratory and economic perspective since it, in theory, can be used for all FXa inhibitors. However, the methods are not validated by the manufacturer or approved for this use. Thus, the responsibility of validating the cutoff limit falls to the end user. Different LMWH/UFH AFXa assays have different sensitivities for apixaban and rivaroxaban [8]. However, our results and previous results indicate that the Innovance LMWH/UFH AFXa assay has sufficient sensitivity and a linear relationship between drug-specific measurements and LMWH/UFH AFXa in the relevant concentration interval, which indicates that drug and method-specific cutoffs can be established [8]. To our knowledge, there has not been a discussion on how laboratories could ensure stable performance for LMWH/UFH AFXa assays to verify low concentrations of DOACs. In our opinion, firstly, validation studies for each combination of drug and method are necessary to establish cutoffs. Secondly, a pragmatic approach could be the verification of each lot/calibration using apixaban and rivaroxaban reference materials (eg, calibrators from other vendors) and daily performance by running apixaban and rivaroxaban controls with acceptance limits specified in IU/L around the established cutoff.

At many hospitals, apixaban and rivaroxaban measurements are not available; an important reason for this is probably the cost or the perceived cost [14]. Even though the number of patients eligible for thrombolysis after drug measurements was small, we believe the benefit more than justifies the cost. Previous studies have found that thrombolysis for AIS may reduce lifetime health costs [18]. While we did not perform a cost-effectiveness analysis, we think that the incremental cost per quality-adjusted life year for drug measurement and thrombolysis compared with no thrombolysis is likely to be well within the limits of willingness to pay off €64 000 used in similar studies in Norway [19]. Since most expenses were from fixed costs such as controls, cost-effectiveness will be higher for centers with a large number of patients. The cost for drug measurement per performed thrombolysis could be lower than reported in our study. With a higher cutoff, more patients will be eligible for thrombolysis. The use of apixaban and rivaroxaban measurement for other indications, both for patients with stroke and other groups, would also lower costs per test. For patients with stroke, dose adjustments or changes of drugs for patients with AIS or in the etiological diagnostic work-up for patients with ICH could be possibilities [20,21]. However, such use does not always necessitate a very short turn-around time; thus, samples could be sent to a centralized laboratory, lowering the costs.

This study has some weaknesses. First, the number of episodes with patients using apixaban/rivaroxaban was limited, and the number of patients treated with thrombolysis was low, meaning that the results may have a high degree of uncertainty. It also restricted us from concluding on the safety of thrombolysis for this patient group. Second, the attending physician in the ED was instructed to order apixaban or rivaroxaban for patients using any of the drugs. This may have been missed for some patients, particularly those with other known contraindications to thrombolysis. Thus, the real number of patient episodes with previous apixaban/rivaroxaban was likely higher. However, since eligibility for thrombolysis is carefully considered for each patient, it is unlikely that patients eligible for thrombolysis after apixaban/rivaroxaban measurement became available were missed. Third, we decided on a conservative low cutoff based on a lack of studies demonstrating the safety of thrombolysis for patients on DOACs. A study published after the end of our project inclusion indicates that it may be safe to treat with thrombolysis at higher DOAC concentrations [10]. However, as already mentioned, this is controversial. Fourth, for the majority of the study period, we used a laboratory-developed method for the measurement of apixaban/rivaroxaban. The method comparison with the *in vitro* diagnostic regulation approved measurement procedures adopted for the last months of the study shown in the Supplementary file demonstrated a bias between the methods. This may have had some importance for the comparison between apixaban/rivaroxaban and LMWH/UFH AFXa measurements. Fifth, in order to evaluate the feasibility of drug measurements in the time-dependent acute setting, we assessed the turn-around time. Samples ordered more than 30 minutes after blood collection were excluded from this analysis.

In conclusion, our study showed that implementation of an SOP for apixaban and rivaroxaban measurements combined with thrombolysis to meet increased acute treatment options in new guidelines was feasible and led to a small increase in the number of patients treated with thrombolysis. However, more studies are needed to establish cutoffs for the safe administration of thrombolysis. Stroke centers, particularly those involved in studies using LMWH/UFH AFXa to verify low drug concentrations, should know and describe the relationship between LMWH/UFH AFXa and drug-specific measurements. We also encourage LMWH/UFH AFXa assay manufacturers to establish apixaban-, rivaroxaban-, and edoxaban-specific cutoffs for their reagents and protocols to ensure stable method performance for this application of the assay.
