# Supplementary material for: Acute ischemic stroke and measurement of apixaban and rivaroxaban: an observational cohort implementation study
Source: Res Pract Thromb Haemost. 2024 Jan 2;8(1):102307. doi: 10.1016/j.rpth.2023.102307 (PMC10837088; doi:10.1016/j.rpth.2023.102307)
Supplement: Supplementary material [file mmc1.docx]

**Supplemental File**

**Table S1: Method specification from September 15 2022 to June 13 2022.** All reagents from Siemens Healthineers unless otherwise stated.

| **Measurand** | **Instrument/**  **application** | **Reagent** | **Calibrator/**  **Traceability** | **Method principle** | **Analytical Precision**  **(CV %)** | **Reportable interval** |
| --- | --- | --- | --- | --- | --- | --- |
| Apixaban | Sysmex  CS-5100/  Application developed for Biophen apixaban method | Innovance anti-factor Xa | Biophen apixaban calibrator (Hyphen biomed) /  Traceable to Hyphen biomed reference standard with concentrations defined by LC-MS/MS | Chromogenic anti-factor Xa activity | <200 µg/L: 10 %  ≥200 µg/L: 5 % | 20-600 µg/L |
| Rivaroxaban | Sysmex  CS-5100/  Application for Innovance anti-factor Xa (Siemens) | Innovance anti-factor Xa | Biophen rivaroxaban calibrator (Hyphen biomed) /  Traceable to Hyphen biomed reference standard with concentrations defined by LC-MS/MS | Chromogenic anti-factor Xa activity | <200 µg/L: 10 %  ≥200 µg/L: 5 % | 20-525 µg/L |
| Heparin  /LMWH | Sysmex  CS-5100/  Application for Innovance anti-factor Xa (Siemens) | Innovance anti-factor Xa | Innovance heparin calibrator/  Traceable to WHO standards for UFH and LMWH | Chromogenic anti-factor Xa activity | <0,60 10^3^U/L: 12 %  ≥0,60 10^3^U/L: 12 % | 0.10-1.50 10^3^U/L |

**Table S2: Method specification from June 14 2022 to September 14 2022.** All reagents from Siemens Healthineers.

| **Measurand** | **Instrument/**  **application** | **Reagent** | **Calibrator/**  **Traceability** | **Method principle** | **Analytical Precision**  **(CV %)** | **Reportable interval** |
| --- | --- | --- | --- | --- | --- | --- |
| Apixaban | Sysmex  CS-5100/  Application for Innovance anti-factor Xa (Siemens) | Innovance anti-factor Xa | Innovance Apixaban Standard/  Traceable to Siemens apixaban reference preparation | Chromogenic anti-factor Xa activity | <200 µg/L: 10 %  ≥200 µg/L: 5 % | 20-350 µg/L |
| Rivaroxaban | Sysmex  CS-5100/  Application for Innovance anti-factor Xa (Siemens) | Innovance anti-factor Xa | Innovance Rivaroxaban Standard/  Traceable to Siemens rivaroxaban reference preparation | Chromogenic anti-factor Xa activity | <200 µg/L: 10 %  ≥200 µg/L: 5 % | 20-350 µg/L |
| Heparin  /LMWH | Sysmex  CS-5100/  Application for Innovance anti-factor Xa (Siemens) | Innovance anti-factor Xa | Innovance heparin calibrator/  Traceable to WHO standards for UFH and LMWH | Chromogenic anti-factor Xa activity | <0,60 10^3^U/L: 12 %  ≥0,60 10^3^U/L: 12 % | 0.10-1.50 10^3^U/L |

**Table S3: Calculations of total costs for apixaban and rivaroxaban measurements (in euros)**

|  | Fixed  yearly costs | Marginal cost  Per analyses | Number of  measurements | Total yearly costs |
| --- | --- | --- | --- | --- |
| Apixaban | 7712 | 3.3 | 124 | 8124 |
| Rivaroxaban | 7712 | 3.3 | 24 | 7792 |

**Figure S1: Method comparison apixaban, see method specifications in Table 1 and Table 2**

**Figure S2: Method comparison rivaroxaban, see method specifications in Table 1 and Table 2**

**Comments on evaluation of apixaban and rivaroxaban measurement methods:**

Analytical performance specifications was based on Gosselin et.al: International Council for Standardization in Haematology (ICSH) Recommendations for Laboratory Measurement of Direct Oral Anticoagulants, Thromb Haemost 2018 Vol. 118 Issue 3 Pages 437-450.

Reproducibility: Coefficient of variation <15 %

Trueness: Bias <15 %

In the concentration interval 20-100 µg/L there was a shift towards 10-20 % lower results for both apixaban and rivaroxaban when we changed methods on June 15^th^ 2022. This change in level would not have affected the clinical decision of any of the patients in the study. It also has a limited effect on the comparisons between apixaban/rivaroxaban and LMWH/UFH AFXa. Performance in external quality assessment (ECAT) have been acceptable for apixaban and rivaroxaban both before and after the change of analytical methods. In general we have had better results compared to the mean of all instrument groups for the new Innovance IVD approved applications from Siemens Healthineers than the previous partly laboratory developed tests.
